# Supplementary material for: Dysphagia Prevalence, Time Course, and Association with Probable Sarcopenia, Inactivity, Malnutrition, and Disease Status in Older Patients Admitted to an Emergency Department: A Secondary Analysis of Cohort Study Data
Source: Geriatrics (Basel). 2021 Apr 26;6(2):46. doi: 10.3390/geriatrics6020046 (PMC8167602; doi:10.3390/geriatrics6020046)
Supplement: Supplementary file 1 [file geriatrics-06-00046-s001.zip › geriatrics-1194577-supplementary.pdf]

Supplemental material 1. Amount and type of missing values in the FAM-CPH study

Paragraph S1.1. Loss at follow-up

Of 127 ED patients included, EAT-10 was completed by *n*=125 at baseline, by *n*=92 at 4-weeks follow-up, and by *n*=65 at 56-weeks follow-up (Figure S1).

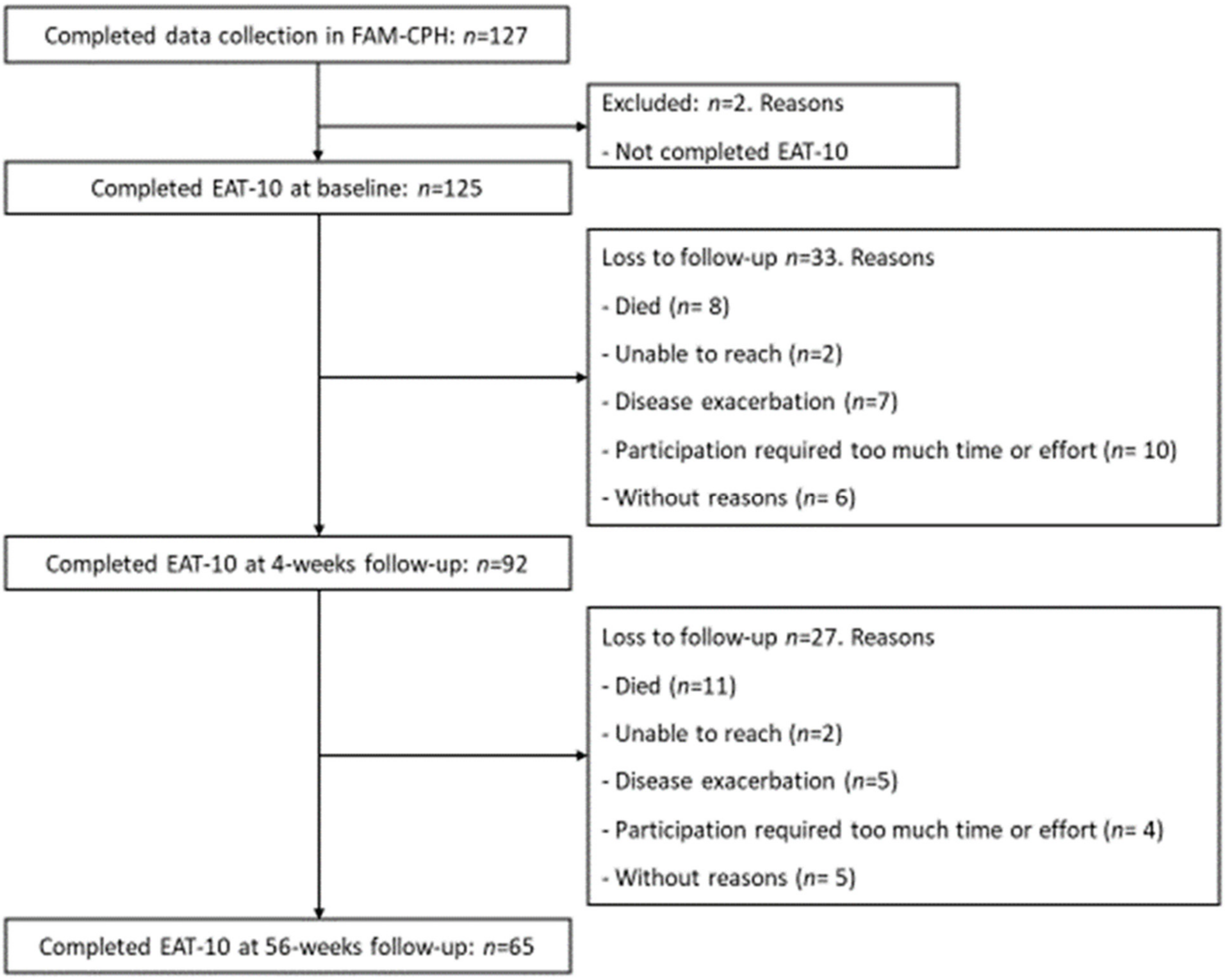

Supplemental Figure S1. Flowchart of inclusion of patients in the secondary analysis.

Paragraph S1.2: Magnitude of missing values

The magnitude of missing values at baseline was relatively high for the 30-CST and the 4MGS, which are two of the parameters for probable sarcopenia. For the 30-CST, a total of *n*=78 (61.4%) had no recorded test results but had a missing code. Of these, there were information related to the initial practice performance for *n*=51 (50.2%). This information involved whether the patient could rise without using the armrest. For the secondary analysis, patients who were able to perform the initial practice performance, but not the test itself were recoded with a score of 0. The reaming patients *n*=27 (21.3%) with missing values but without information from the initial practice performance were kept as missing (Table S1). For the 4MGS, a total of *n*=44 (34.6%) had no recorded test results but had a missing value. For the secondary analysis, patients who were recorded as habitually with no gait function were recoded with a score of 0 (*n*=4) and the reaming patients *n*=40 (31.5%) were kept as missing (Table S1). For both variables, the coded reasons for the remaining missing values were mainly related to inability to participate due to physical symptoms.

Supplemental material Table S1. Missing value pattern of data included in the secondary analysis

	**Baseline**		**4-weeks follow-up**	**56- weeks follow-up**
	**Total sample** *n* (%) of 127	**Complete EAT-10** *n* (%) of 125	**Complete EAT-10** *n* (%) of 92	**Complete EAT-10** *n* (%) of 65
PRIMARY OUTCOME				
**Swallowing function**				
EAT-10	2 (1.6 %)	0 (0.0 %)	0 (0.0 %)	0 (0.0 %)
SECONDARY OUTCOMES				
**Parameters for probable sarcopenia**				
HGS	3 (2.4 %)	3 (2.4 %)	5 (5.4 %)	2 (3.1 %)
30-CST	27 (21.3 %)	26 (20.8 %)	4 (4.3 %)	2 (3.1 %)
4MGS (m/s)	40 (31.5 %)	40 (31.5 %)	7 (7.6 %)	4 (6.2 %)
**Activity status**				
SNAQ	3 (2.4 %)	2 (1.6 %)	0	2 (3.1 %)
FRS	3 (2.4 %)	2 (1.6 %)	4 (4.3 %)	4 (6.2 %)
**Nutritional status**				
BMI	0	0	0	0
MNA-SF	3 (2.4 %)	2 (1.6 %)	1 (1.1 %)	1 (9.8 %)
**Disease status**				
Admission diagnoses	5 (3.9%)	5 (4.0%)	NC	NC
CCI	8 (6.3%)	8 (6.4%)		
CRP (mg/L)	0	0	9 (9.8 %)	17 (26.2 %)
suPAR (ng/mL)	3 (2.4 %)	3 (2.4 %)	11 (12.0 %)	17 (26.2 %)
TNF-α (pg/mL)	7 (5.5 %)	6 (4.8 %)	10 (10.9 %)	6 (9.2 %)
IL-6 (pg/mL)	7 (5.5 %)	6 (4.8 %)	10 (10.9 %)	6 (9.2 %)

Abbreviations: EAT-10, Eating Assessment Tool; HGS, handgrip strength; 30-CST, 30 seconds chair stand test; 4MGS, 4-meter gait speed; SNAQ, Simplified Nutritional Appetite Questionnaire; FRS, Functional Recovery Score; MNA-SF, Mini Nutritional Assessment Short Form; BMI, Body Mass Index; CCI, age-adjusted Charlson comorbidity index; NC, not computed since admission diagnosis are only presented at baseline and CCI is only calculated for baseline data; CRP, C-reactive protein; suPAR, soluble urokinasee plasminogen activator receptor; TNF-α, tumor necrosis factor-α; IL-6, interleukin-6.

Paragraph S1.3. Type of missingness

To determine whether to use imputation methods, the type of missingness (random or not) was investigated using multiple logistic regression analysis with the missingness variable as dependent variables (recoded into 0=not missing and 1=missing) and the demographics and selected secondary outcomes as the independent variables. This is presented in Table S2.

Supplemental material Table S2. Association of missing values of 30-CST and 4MGS and demographics and secondary outcomes.

	30-CST		4MGS	
	OR (95% CI)	*p*	OR (95% CI)	*p*
Age	0.95 (0.89-1.02)	0.182	0.97 (0.91-1.04)	0.433
Gender	1.37 (0.49-3.84)	0.549	1.33 (0.48-3.73)	0.583
OMC-score	1.01 (0.90-1.13)	0.872	0.95 (0.86-1.05)	0.305
MNA-SF score	0.91 (0.73-1.15)	0.443	0.84 (0.68-1.05)	0.124
SNAQ-score	1.03 (0.83-1.28)	0.782	1.00 (0.81-1.21)	0.917
FRS-score	0.96 (0.94-0.99)	0.003	0.95 (0.92-0.98)	0.000

30-CTS, 30 seconds chair stand test; 4MGS, 4-meter gait speed; OMC, Orientation Memory Concentration-test; MNA-SF, Mini Nutritional Assessment Short Form, SNAQ, Simplified Nutritional Appetite Questionnaire; FRS, Functional Recovery Score.

For both missingness variables (30-CST and 4MGS), declined functional performance status (FRS) was a significant predictor ($p<0.05$) for a missing value (Table S2). It was therefore concluded that the data were not missing completely at random but might be dependent on the variables themselves. Additionally, several of the variables in the dataset were non-normally distributed as measured by the Kolmogorov-Smirnov test. Therefore, imputation was not applied, and all analyses were performed as complete case analyses.
